# A Case of Recurrent Skin Abscesses: A Conundrum Solved after Obtaining a Thorough Sexual History

**DOI:** 10.1155/2017/4765697

**Published:** 2017-03-13

**Authors:** Diego P. Peralta, Aymara Y. Chang, Enrique Soto-Ruiz

**Affiliations:** ^1^Division of Infectious Diseases, Texas Tech University Health Sciences Center El Paso Paul L. Foster School of Medicine, El Paso, TX 79905, USA; ^2^Division of General Internal Medicine, Texas Tech University Health Sciences Center El Paso Paul L. Foster School of Medicine, El Paso, TX 79905, USA; ^3^Department of Pharmacy, University Medical Center of El Paso, El Paso, TX 79905, USA

## Abstract

*Background*. Despite the improvement in patient-physician communication techniques, sexuality and sexual health continue to be challenging areas for discussion during a clinical encounter. Most people are not prepared to discuss sexual matters openly as it can be perceived as negative or inappropriate. Consequently, an incomplete health assessment can result in delayed diagnosis or misdiagnosis.* Case Report*. We present a 33-year-old woman who developed recurrent left breast abscesses. She required multiple incision and drainage procedures in the operating room followed by antimicrobial therapy. Although she always had an initial improvement with this approach, she continued to have recurrences and development of new abscesses in other body areas. The polymicrobial nature of her recurrences prompted an extensive and costly workup to determine the nature of her condition. The cause was finally elucidated when a thorough sexual history was obtained. Poor hygiene practices during her sexual encounters were considered the cause of her recurrent abscesses. After medical therapy and modification of her sexual practices, she has not developed new recurrences for more than two years.* Conclusion*. Discussions on sexuality and sexual health are important parts of any clinical encounter, yet frequently forgotten or avoided. Becoming aware of their importance would avoid delayed diagnosis or misdiagnosis.

## 1. Background

Sexuality and sexual health are subjects commonly overlooked by physicians despite being important parts in the health assessment of a patient [1–3]. Multiple reasons have been attributed to this omission including barriers in doctor-patient communication, time restrictions, patient's embarrassment and negative perception, physician's lack of training, and uncertainty of risk related and medical relevance [3–5]. Although new interview techniques have been advocated, such as using inclusive questions and language during sexuality or sexual health discussions [5, 6], their application is still insufficient. Laverse et al. [7] describe two cases of sexually transmitted infections, which lacked an initial collection of sexual history and resulted in detrimental misdiagnosis and delayed diagnosis. A complete sexual history provides valuable information regarding risk factors for sexually transmitted infections (STIs), sexual orientation, libido problems, and preferred sexual practices among others [3, 5].

We report a woman who developed recurrent polymicrobial skin abscesses likely resulting from her sexual practices. The origin of her illness was only elucidated when a detailed sexual history was obtained.

## 2. Case Report

A 33-year-old woman was admitted to our institution for evaluation of recurrent left breast abscesses. Her medical history included postpartum depression and an abnormal cervical cytology treated with loop electrosurgical excision procedure. There was no personal nor family history of immunodeficiency conditions. She was an occasional alcohol drinker and a daily marijuana smoker. She worked as a nursing assistant at a local nursing home. She denied sick contacts, recent travel, and animal or insect exposure.

She had six previous admissions within the past two months with left breast abscesses for which she underwent incision and drainage in the operating room (OR), followed by antimicrobial therapy. During this admission, she reported a recurrence of left breast abscesses and development of new abscesses in her buttocks. Her review of systems was otherwise negative.

**Figure 1:**
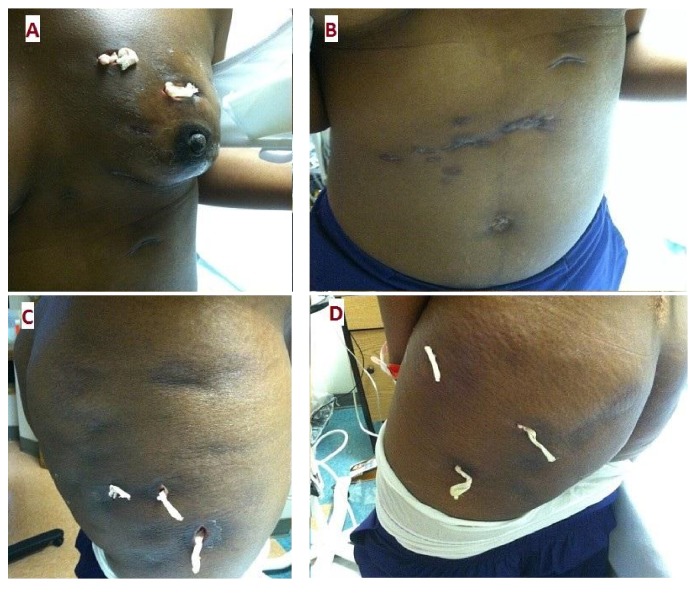
Distribution of skin abscesses. (A) Left breast. (B) Abdomen. (C) Buttocks. (D) Thighs.

Physical examination showed a woman in distress with a blood pressure of 108/67 mmHg, a pulse of 103 beats per minute, a temperature of 36.6°C, and a respiratory rate of 17 breaths per minute. The left breast had a visible deformity and asymmetry compared to the right side. Multiple scars and packed wounds were seen (Figure 1(A)). There was no nipple discharge, retraction, or peau d'orange skin appearance. Induration and fluctuation were palpated; however, discomfort limited a further examination. In the abdominal area, she had keloid-like scars resulting from old skin abscesses (Figure 1(B)). Her buttocks and thighs had several areas of hyperpigmentation and dimpling (Figures 1(C) and 1(D)). Palpation of these zones demonstrated induration, fluctuation, warmth, and tenderness. The external genitalia and perineal area examination did not reveal mucosal or skin lesions.

Intravenous vancomycin 1500 mg every 12 hours, cefepime 2 g every 8 hours, and oral metronidazole 500 mg every 8 hours were initiated as an empiric antimicrobial regimen. Plastic surgery service decided to perform left breast and buttocks incision and drainage procedures in the OR. New specimens were collected from both left breast and buttocks for culture and histopathology, respectively.

At this point, there was uncertainty as to the nature of her condition. Infectious and noninfectious causes, such as immunodeficiency syndromes and malignancy, were hypothesized. Initial bloodwork showed leukocytosis with left shift, normal chemistry panel and hemoglobin A1C, nonreactive rapid plasma reagin, negative* Treponema pallidum* particle agglutination assay, and elevated sedimentation rate and C-reactive protein. Urine nucleic acid amplification testing for* Neisseria gonorrhoeae* and* Chlamydia trachomatis* were negative. They were performed in a Panther® System (Hologic, Marlborough, USA). Additional workup yielded normal levels of complement and immunoglobulins (e.g., IgA, IgE, IgG, and IgM), normal CD4+ cell count, and negative fourth-generation HIV antigen/antibody combo test, which was performed in an ARCHITECT* i*2000SR® Immunoassay Analyzer (Abbott, Abbott Park, USA). For hepatitis B and C serologic evaluation a VITROS® ECiQ Immunodiagnostic System (Ortho Clinical Diagnostics, Raritan, USA) was used. It yielded negative hepatitis B core antibody and positive surface antibody and negative hepatitis C antibody.

Cultures from both breast tissue and buttocks yielded similar Gram-negative bacteria to previous admissions, for example,* Citrobacter amalonaticus, Klebsiella pneumoniae, Bacteroides fragilis, Pseudomonas aeruginosa,* and* Enterococcus avium*. Also, methicillin-resistant* Staphylococcus aureus*, *β*-hemolytic group F streptococci,* Viridans streptococci*, and* Candida albicans* were isolated in one of the buttocks specimens.* Acinetobacter baumannii* grew in one set of blood cultures. Urine and stool cultures were not collected as patient never manifested gastrointestinal or urinary symptoms. The histopathologic evaluation showed a benign breast tissue with abscess formation and necrosis. Acid-fast bacilli, Gomori methenamine silver, and pancytokeratin immunohistochemical stains were negative for pathogens and atypical epithelial cell population, respectively.

Each surgical specimen underwent routine bacterial workup. For aerobes, Sheep Blood, Chocolate, and MacConkey agars were used and, for anaerobes, Anaerobic Sheep Blood and Kanamycin Vancomycin Blood agars CDC. Agars used are from Remel Microbiology Products (Thermo Scientific, Lenexa, USA). Both aerobic and anaerobic cultures were maintained at a temperature of 35°C (+/−2°C) during the incubation period. Chocolate and Sheep Blood agars were incubated with 5% carbon dioxide in a Lab-Line IIi CO_2_ incubator (Thermo Scientific Barnstead, Massachusetts, USA) whereas the MacConkey agar was incubated in a 6M Incubator (Precision Scientific, Illinois, USA). The anaerobic cultures were enclosed and remained in an anaerobic chamber during the incubation period. Bacterial growth was evaluated and documented every 12 hours for the aerobic cultures and every 48 hours for the anaerobic cultures. On the other hand, blood cultures were processed in a BACTEC™ 9240 Blood Culture System (Becton, Dickinson and Company, Franklin Lakes, USA). A VITEK® 2 System (bioMérieux, Marcy-l'Étoile, France) was used for microbial identification and antibiotic susceptibility testing of positive specimens.

Considering the predominance of Gram-negative bacteria in cultures and development of new abscesses in the proximity of the rectal area, a gastrointestinal (GI) cause was contemplated. Therefore, imaging studies were ordered to evaluate intra-abdominal pathologies. However, computerized tomography scans and a magnetic resonance enterography (MRE) failed to demonstrate abnormalities.

On the other hand, being a nursing assistant and her history of postpartum depression were brought up as possible risk factors for a psychiatric illness as the cause of her repetitive abscesses. However, a comprehensive evaluation did not find an association between her clinical presentation and a psychiatric disorder.

While receiving antimicrobial therapy, her condition improved and bacteremia resolved. Nevertheless, the etiology of the repetitive abscesses remained uncertain. Frequent interviews were performed to obtain new clues to explain her illness. Areas previously omitted including a complete sexual history were discussed with the patient. After several conversations, she finally became comfortable enough to reveal that she was a lesbian, had a stable relationship with the same female partner for almost a year, and practiced receptive anal intercourse by introducing a sex toy or her partner's fingers. Further questioning revealed that her partner frequently kissed, licked, touched, nipped, and scratched multiple areas of her body, including breasts and buttocks, without a proper hand washing or sex toy cleaning.

After knowing these facts, the patient and her partner were extensively counseled about safe sexual practices and appropriate hygiene to prevent recurrences. She received two weeks of inpatient intravenous vancomycin, cefepime, and metronidazole followed by one week of outpatient oral trimethoprim/sulfamethoxazole 800/160 mg twice daily, levofloxacin 750 mg daily, and metronidazole 500 mg every 8 hours along with aggressive wound care. Antifungal therapy was not used since* Candida albicans* was considered a colonizer. After completing the antimicrobial therapy and changing her sexual practices, she has not developed recurrences for more than two years.

## 3. Discussion

Our patient was admitted for evaluation and treatment of repetitive abscesses, which were thought to have an organic cause leading to an extensive and costly workup. Although many efforts were made to obtain pertinent information to make a diagnosis, it was not until several days later that we discovered the cause. This case shows the complexity and significance in getting sexually related information, that is, sexual history, despite advances in doctor-patient communication techniques [3, 6, 8, 9].

Discussing sexuality and sexual health continues to be a commonly omitted area, yet a critical part of the health assessment of a patient [1–3]. In fact, sexual history provides a vast amount of useful information, for example, risk factors for STIs, sexual orientation, libido problems, and preferences in sexual practices [3, 5]. In the case of our patient, a detailed sexual history allowed us to ascertain the cause of her skin abscesses and prevent further recurrences.

Multiple challenges should be overcome to obtain an accurate sexual history including barriers in communication, time constraints, embarrassment, lack of training, and uncertainty of risk related and medical relevance [3–5]. In our case, getting over those challenges facilitated the gathering of essential information to solve this conundrum. Most people are not prepared to discuss sexual matters openly as it can be perceived as negative or inappropriate. Use of inclusive questions and language is advocated while discussing sexuality or sexual health to facilitate the doctor-patient communication. Examples of inclusive questions are as follows: “do you have sex with men, women, or both?” or “are you currently involved in a sexual relationship?” These questions should not make assumptions, for example, asking a female patient: “do you have a boyfriend?” or asking a male patient: “when did you first become interested in girls?” [3, 6, 8].

Besides communication issues associated with our patient's sexuality, this case was unique due to the nature of the infection and its unexpected outcome. Her presentation with predominant Gram-negative bacteria abscesses prompted an extensive and costly workup as Gram-positive bacteria are the most common cause of soft tissue infections [10, 11]. When other pathogens, such as Gram-negative bacteria or fungi, are isolated, they are frequently associated with chronic infections, prior long-term antimicrobial therapy, surgical site infections of the abdominal wall, soft tissue infections with reduced vascular perfusion, infections in the anal and perineal region, or neutropenia [10]. Atypical infections, psychiatric disorders, and immunodeficiency and malignant conditions were considered as the cause of her recurrences. However, the complete assessment yielded negative results.

On the other hand, a disorder of the GI tract such as inflammatory bowel disease might present with extraintestinal manifestations in up to 40% of cases [12]. The skin is one of the most commonly affected organs in these cases. In Crohn's disease (CD), cutaneous manifestations occur more frequently in adult females with established intestinal disease. Cutaneous CD usually affects the lower extremities (38%), abdomen and trunk (24%), upper extremities (15%), face and lips (11%), and intertriginous areas (8%) [12]. Erythema nodosum, pyoderma gangrenosum, aphthous stomatitis, and neutrophilic dermatoses are the typical skin findings of cutaneous CD. Further, perianal CD including erythema, abscesses, ulcers, fissures, and fistulas occur in about 50% of CD patients at some point during their clinical course [12]. Our patient never had clinical manifestations of CD, such as diarrhea, bloody stools, abdominal pain, or weight loss. MRE and histopathologic evaluation of breast tissue failed to demonstrate findings consistent with IBD or other GI processes.

We hypothesized that the polymicrobial nature of the skin abscesses was the result of microbial inoculation in her breast and gluteal area during her sexual encounters. The Gram-positive bacteria were likely coming from the skin and oral flora whereas the Gram-negative bacteria from the GI tract. Isolated* Candida albicans* was also probably coming from the GI tract or the result of overgrowth secondary to prior antimicrobial use. Polymicrobial inoculation likely occurred during kissing, licking, and contact with partner's artificial fingernails and sex toys. Multiple small skin lacerations caused by the partner's fingernails were probably the infection port of entry. We believe that different pathogens became colonizers of both partner's fingernails and sex toys due to poor hygienic sexual practices. Artificial fingernails have been previously linked to bacterial transmission, for example,* Pseudomonas aeruginosa* [13, 14] and* Serratia marcescens* [15]. Sex toys have been associated with the transmission of STIs such as trichomoniasis, genital herpes, human papillomavirus [16], bacterial vaginosis [17], and human immunodeficiency virus [18].

We support our hypothesis by a negative workup for underlying medical conditions, such as a psychiatric condition, immunosuppression, GI pathology, or malignancy and resolution of patient's symptoms and recurrences after behavior modification of her sexual practices. In fact, there have not been reports of new episodes for more than two years.

## 4. Conclusion

Multiple barriers are encountered while discussing sexuality and sexual health with patients. These obstacles can be overcome with an awareness of their importance and practice. Therefore, we recommend including a discussion about sexuality and sexual health in every clinical encounter to improve patient care and avoid delayed diagnosis and misdiagnosis. Our patient is an example of the importance of a detailed sexual history acquisition.
